# New Insights into the Microbiota of the Svalbard Reindeer *Rangifer tarandus platyrhynchus*

**DOI:** 10.3389/fmicb.2016.00170

**Published:** 2016-02-23

**Authors:** Sylwia Zielińska, Dorota Kidawa, Lech Stempniewicz, Marcin Łoś, Joanna M. Łoś

**Affiliations:** ^1^Department of Molecular Biology, University of GdańskGdańsk, Poland; ^2^Department of Vertebrate Ecology and Zoology, University of GdańskGdańsk, Poland

**Keywords:** bacterial community, reindeer feces, 16S rDNA, non-invasive method, Arctic, Svalbard reindeer

## Abstract

Svalbard reindeer (*Rangifer tarandus platyrhynchus*) is a non-migratory subspecies of reindeer inhabiting the high-arctic archipelago of Svalbard. In contrast to other *Rangifer tarandus* subspecies, Svalbard reindeer graze exclusively on natural sources of food and have no chance of ingestion of any crops. We report the use of a non-invasive method for analysis of fecal microbiome by means of sequencing the 16S rDNA extracted from the fecal microbiota of *R. tarandus platyrhynchus* from a small, isolated population in Hornsund, South Spitsbergen National Park. Analyses of all samples showed that 99% of the total reads were represented by Bacteria. Taxonomy-based analysis showed that fecal bacterial communities consisted of 14 phyla. The most abundant phyla across the population were *Firmicutes* and *Bacteroidetes*, and those phyla jointly accounted for more than 95% of total bacterial sequences (ranging between 90.14 and 98.19%). Specifically, *Firmicutes* comprised 56.53% (42.98–63.64%) and *Bacteroidetes* comprised 39.17% (34.56–47.16%) of the total reads. The remaining 5% of the population reads comprised of *Tenericutes, Cyanobacteria*, TM7, *Actinobacteria, Proteobacteria, Verrucomicrobia, Elusimicrobia, Planctomycetes, Fibrobacteres, Spirochaetes, Chloroflexi*, and *Deferribacteres*. Differences in the fecal bacteria composition between particular reindeer were not statistically significant which may reflect the restricted location and similar diet of all members of the local population.

## Introduction

Previously, microbial community inhabiting reindeer gut was mainly studied by traditional cultivation methods or classical molecular techniques (Mathiesen et al., 1987; Sørmo et al., 1994; Aagnes et al., 1995; Sundset et al., 2007, 2009; Glad et al., 2014) resulting in insufficient characterization. Recent advances in high-throughput sequencing technologies provide access to culture-free characterization of microbial community structures in variety of environments. Next Generation Sequencing (NGS) provides an opportunity to analyze low abundance microbial components that contribute to less than 1% of the total population (Lee et al., 2012). Data obtained with traditional approaches are a valuable source, but cannot be directly compared with the NGS data, which provides a possibility to detect the vast majority of bacteria and can show the real contribution of a given species to the whole bacterial community. It is not surprising that data obtained in a traditional way may underestimate the number of certain bacteria, especially since the majority of 16S rRNA genes sequenced from reindeer gut represent novel and uncultured bacterial species (Sundset et al., 2007). It has been estimated that using culture based techniques (isolation, enumeration, and nutritional characterization) can only account for 10–20% of the total rumen microbial community (Makkar and McSweeney, 2005). Furthermore, according to Ramsak et al. (2000) ruminal *Bacteroides* are seriously under-represented in cultivation based methods and among cultured isolates, which could be due to their stricter requirement for anaerobic cultivation.

In most studies, samples for bacterial taxonomic identification are taken from rumen (Makkar and McSweeney, 2005; Sundset et al., 2007; Lee et al., 2012; Glad et al., 2014). It seems reasonable, as the rumen fermentation is a key element in the process of digestion (Gruninger et al., 2014). There are ~10^11^ microbial cells per gram of rumen content, with bacteria, archaea, fungi, ciliate protozoa, and viruses being among them (Henderson et al., 2013). However, taking rumen samples can implicate the death of the animal or at least, cause the animal considerable stress, especially in the case of wild animals not accustomed to humans. Clearly, in some cases, individuals are sacrificed in accordance with specific country regulations (Sundset et al., 2009). That kind of practice during sampling prevents tracking changes of rumen microbiota composition over time e.g., according to seasonal environmental, and ecological changes, including food availability. Rumen content can be also collected from animals during the experiment periodically (e.g., from rumen-fistulated animals; Lee et al., 2012; Glad et al., 2014), but that makes it more expensive, time consuming, and uncomfortable for the studied subject. Moreover, such collecting methods interfere with natural animal environment, especially when studying wild populations with limited or no previous exposure to humans, for example in National Parks. In such situations, testing fecal samples can be a good alternative, especially when cyclical nature of the research and examining dietary changes are considered as a subject of the study (Fernandes et al., 2014). That kind of approach is non-invasive and provides a low level of interference in natural populations, practically eliminating additional environmental stress. This method has also some disadvantages like e.g., a risk of contamination, but well planned sampling method minimizes these risks.

The molecular diversity of the rumen microbiome has been studied in two subspecies of reindeer: Svalbard reindeer (*Rangifer tarandus platyrhynchus*) in central Spitsbergen, and nominative subspecies reindeer (*R. tarandus tarandus*) in northern Norway (Sundset et al., 2007, 2009). Reindeer are foregut fermenters whose digestion depends on a symbiotic association with the complex microbiota resident within their rumen (Mathiesen et al., 2005). Svalbard reindeer live under extremely severe environmental and nutritional conditions in the high Arctic, where snow may cover the vegetation from September until the end of May (Alendal et al., 1979). Their diet, characterized by a high variety of arctic plants, resulted in a unique rumen microbial ecosystem (Sundset et al., 2007, 2009). They prefer vascular, easily digestible plants. During summer, they selectively graze on *Oxyria digyna*, supplemented with *Saxifraga nivalis, Saxifraga oppositifolia, Pedicularis dasyantha, Pedicularis hirsuta, Bistorta vivipara, Brassicaceae*, and *Cyperaceae* (Bjune, 2000; Lindner, 2003). Svalbard reindeer population is mainly distributed in Spitsbergen, the largest island of the Svalbard archipelago. Contrary to the continental *Rangifer* subspecies, Svalbard reindeer do not perform seasonal migrations. Radio-collared females travel less than 0.7 km per day, on average, in both summer and winter (Tyler and Øritsland, 1990). Individuals are solitary or live in/form small groups (Tyler and Øritsland, 1989). In the Hornsund area they have been observed regularly beginning in the 1990s (Fossa et al., 2002), however, there are not many individuals in the area. This particular subspecies comprises the northernmost population of *Rangifer* inhabiting larger islands of the Svalbard archipelago, where it is found in almost all non-glaciated areas, mainly in the strand flats and valleys (Aanes, 2005). The coastal terraces of the Hornsund area are almost uninhabited by humans. The only permanent settlement is a Polish Polar Station, where nine to ten people spend a whole year. Additionally, during summer (July–August) small groups of scientists and tourists stay there for short periods of time.

Our study provides a comprehensive view of the fecal microbiota of the Svalbard reindeer from Hornsund (SW Spitsbergen), analyzed by the NGS method, which most of all allowed to overcome the limitations of classical approaches and analysis of the low abundance microbial components. Acquisition of such information might be of high importance to reindeer breeders due to economic aspects. For all circumpolar indigenous peoples in Eurasia and North America, wild and semi-domesticated reindeer are an important source of clothing, shelter, tools and food, especially since reindeer meat and milk, as well as internal organs, can be eaten as elements of traditional dishes. Additionally, sale of fur and meat is an important source of their income.

## Materials and methods

### Sample collection

**Figure 1:**
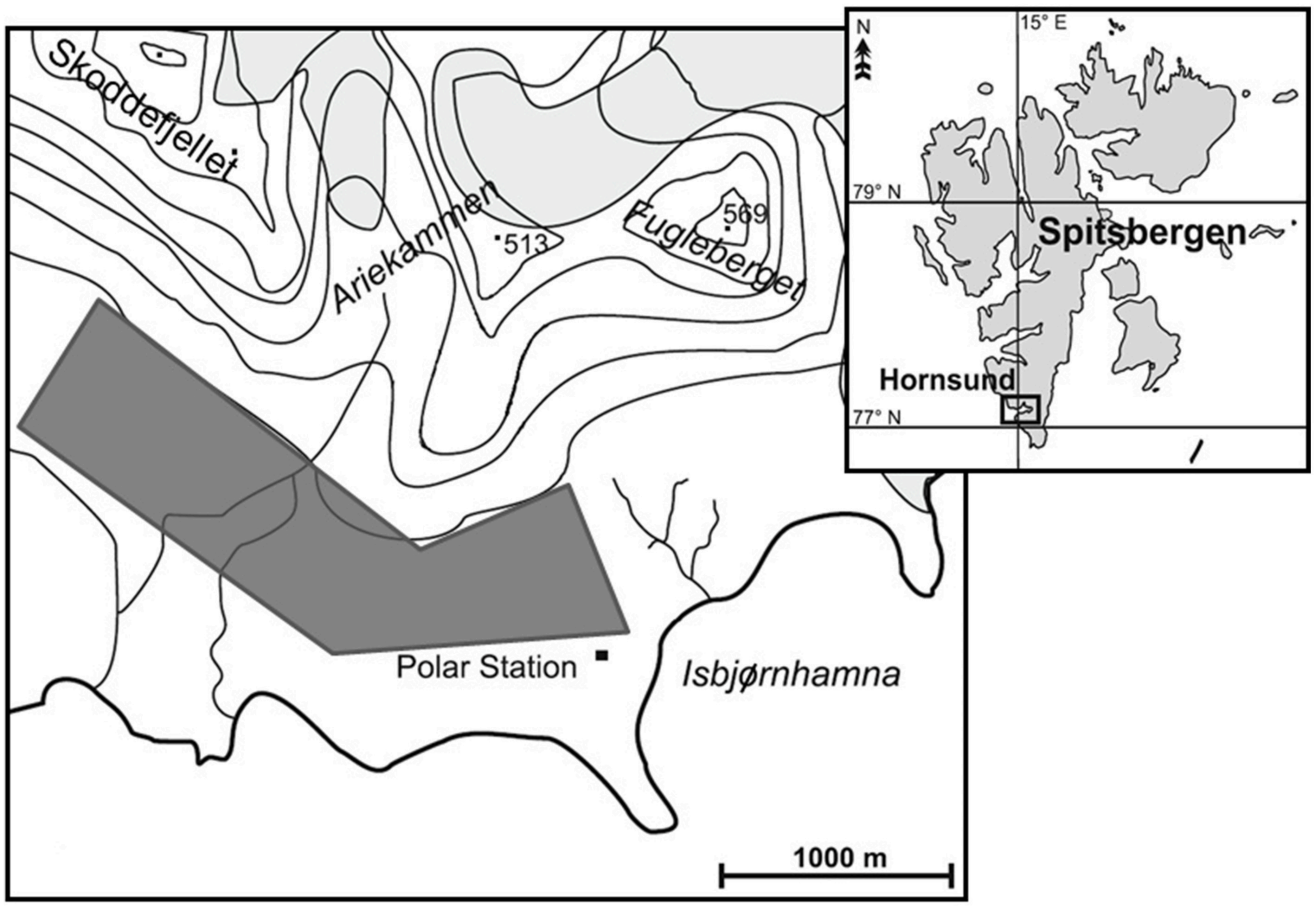
**Maps of the study area**. Gray shade indicates the reindeer's grazing area where the individuals were observed and the fecal samples were collected.

Ten fecal samples (R1–R10) were collected in the northern part of the Hornsund fiord (SW Spitsbergen; 77° 0′N, 15° 33′E), in the flat area between the seashore and the Ariekammen and Skoddefjellet mountains (Figure 1). Reindeer were followed until they produced a fresh portion of feces, which were collected, stored separately in plastic bags, and frozen at −20°C within max. 30 min. Five samples (R3, R5, R6, R8, and R10) were from five different adult males. The rest of the samples (R1, R2, R4, R7, R9) were collected in the area where three females and two young individuals (more than 1 year old) were present, however, they could not be clearly associated with a given individual (we could not be absolutely certain that all those samples were from different individuals). This was due to a very close proximity of individuals in this group. Samples were collected in the late summer of 2013, between July 26th and August 5th, which corresponds to the period when the food nutrient content and reindeer digestibility are in peak (Larsen et al., 1985; Mathiesen et al., 2005).

### DNA extraction

After thawing, samples were homogenized using the MP FastPrep-24 Instrument (MP Biomedicals Inc.). Nucleic acids were extracted from 200 mg of feces by the GeneMATRIX Stool DNA Purification Kit (Eurx Ltd.) and stored at −20°C before further use. To avoid cross contamination of samples, the whole process was performed using sterile equipment. The quantity and quality of the extracted DNA were evaluated by using a Nano Drop spectrophotometer and agarose gel electrophoresis.

### Bacterial 16S rDNA amplification and sequencing

The V3–V4 hypervariable regions of bacterial 16S rDNA region were amplified using the following primer set: 341F - CCTACGGGNGGCWGCAG and 785R - GACTACHVGGGTATCTAATCC. The targeted gene region has been shown to be the most appropriate for the Illumina sequencing (Klindworth et al., 2013). Each library was prepared in a two-step PCR protocol based on Illumina's “16S metagenomic library prep guide” (15044223 Rev. B) using NEBNext® High-Fidelity 2xPCR Master Mix (New England BioLabs) and Nextera Index kit (2 × 250 bp). Paired-end (PE, 2 × 250 nt) sequencing, with a 5% PhiX spike-in, and was performed on an Illumina MiSeq (MiSeq Reagent kit v2) following manufacturer's run protocols (Illumina, Inc., San Diego, CA, USA), at Genomed, Warsaw, Poland. The automatic primary analysis and the de-multiplexing of the raw reads were performed on MiSeq with the use of MiSeq Reporter (MSR) v2.4 (BaseSpace).

### Processing of sequencing data and statistical analysis

Samples were processed and analyzed using the Quantitative Insights Into Microbial Ecology (Qiime) pipeline v 1.8.0 (Caporaso et al., 2010) software. Low quality PE reads were removed before further analysis and quality-filtered reads were merged based on the overlap of PE read with the use of fastq-joint (Aronesty, 2011). The remaining sequences that did not meet quality criteria were removed from further analysis. Clustering of operational taxonomic units (OTUs) was performed at 97% similarity by using the uclust method (Edgar, 2010). OTUs were assigned to taxa using GreenGenes v13_5 as the reference (McDonald et al., 2012). The chimera sequences were detected with the use of a tool called Chimera Slayer (Haas et al., 2011). Based on clusters, the diversity indices were estimated, including the Chao1, Shannon, and Simpson indices. Also, for some additional data analysis and visualization, independent analyses were performed with the use of BaseSpace Application 16S Metagenomics v1.0 (Illumina, INC.). NGS data are deposited and fully available under study accession number PRJEB8619 on ENA—European Nucleotide Archive and BaseSpace Application (myillumina, public data) under project name: Study of fecal microbiota of Svalbard reindeer, Hornsund, Southwest Spitsbergen or under URL link https://basespace.illumina.com/s/e6vVqtk34KNe.

The average abundance of bacterial 16S rDNA sequences at each phylum level was calculated for the total population (Rt). Similarity percentage (SIMPER) analysis was performed to calculate the average dissimilarities in microbial community structures between the samples, and to assess which phylum was responsible for the observed differences. Hierarchical clustering based on Bray-Curtis similarity index was performed. The differences in microbial community structures were tested by the χ2 test (with Bonferroni correction in case of multiple pairwise comparisons). Statistical analyses were performed using PAST 3.0 software (Hammer et al., 2001; SIMPER and cluster analysis) and STATISTICA 10.0 (χ2 test).

## Results

### General description of sequencing results

In the material isolated from all reindeer fecal samples, we obtained 380,849 good quality 16S rRNA gene sequences (V3–V4 region). On average, we obtained 38,085 (in the range of 22,997–54,042) paired sequences per sample. Generally, 15,000–100,000 reads per sample are sufficient for classification, as described in Illumina 16S Metagenomic Sequencing Protocol. Table 1 lists the number of OTUs and the diversity index for each member of the population (R1–R10) and for the total population (Rt). Principal coordinate analysis, performed to compare the apparent compositions of microbial communities among individuals, is presented in Supplementary Figure 2. We were able to classify all obtained sequences at the phylum level. Detailed taxonomic analyses on different ranks are available in supplementary data as sunburst charts for each individual, as well as for total population (Supplementary Figure 1) and are also summarized in Supplementary Table 1.

**Table 1:** **Summary of the sequencing data and statistical analysis of bacterial microbial communities**.

**ID**	**No. of bacterial reads**	**Average length (bp)**	**No. of observed OTUs**	**Chao1 index**	**Shannon index**	**Simpson index**
R1	23,166	449	847	1115	6.30	0.94
R2	35,775	449	887	1022	6.09	0.93
R3	35,302	449	921	1093	6.41	0.96
R4	36,733	449	931	1084	6.55	0.96
R5	54,042	449	979	1030	6.53	0.96
R6	45,210	450	976	1070	6.30	0.94
R7	47,996	449	1002	1111	6.44	0.95
R8	22,997	449	800	1105	5.98	0.92
R9	30,239	451	833	1044	5.98	0.93
R10	49,389	454	904	973	6.39	0.95
Rt	380,849	450	1883	1065	6.30	0.94

*The ID abbreviations are defined in text. The number of OTUs (operational taxonomic units) was generated at the 97% sequence similarity cut-off. Diversity indices represent the randomly selected subsets for each sample normalized to 25378 sequences*.

### Bacterial community composition

**Figure 2:**
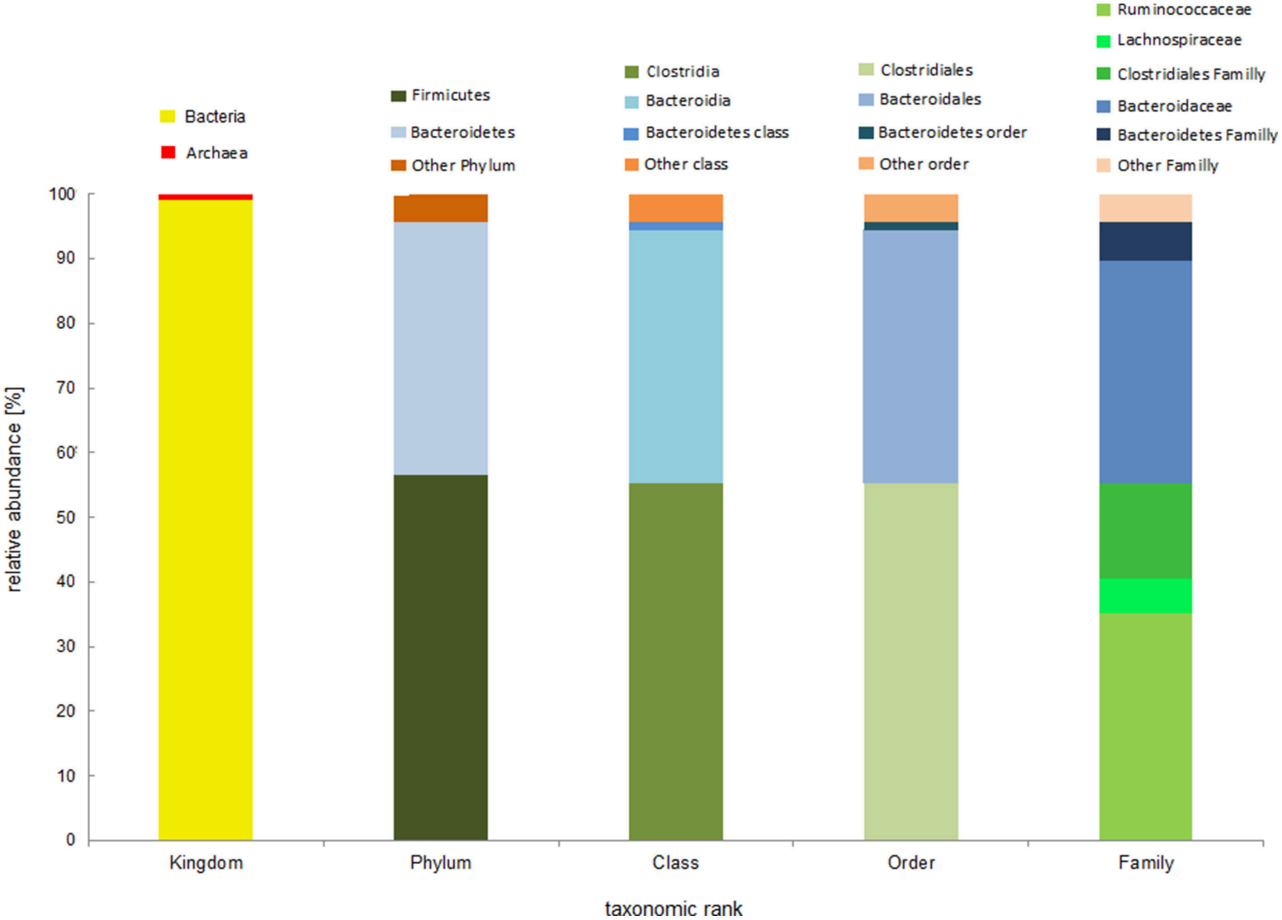
**Abundance of bacterial 16S rDNA sequences in total population**. Column chart shows the relative abundance of the top classification results at different taxonomic levels. The first column represents the total reads for *bacteria* and *archaea*. Next columns represent the total reads for *bacteria*. At different taxonomic levels, “other” corresponds to bacteria with only a small share in the total population and to those unidentified at a particular taxonomic rank. Detailed taxonomic analyses on different ranks are available in supplementary data (Supplementary Figure 1).

**Figure 3:**
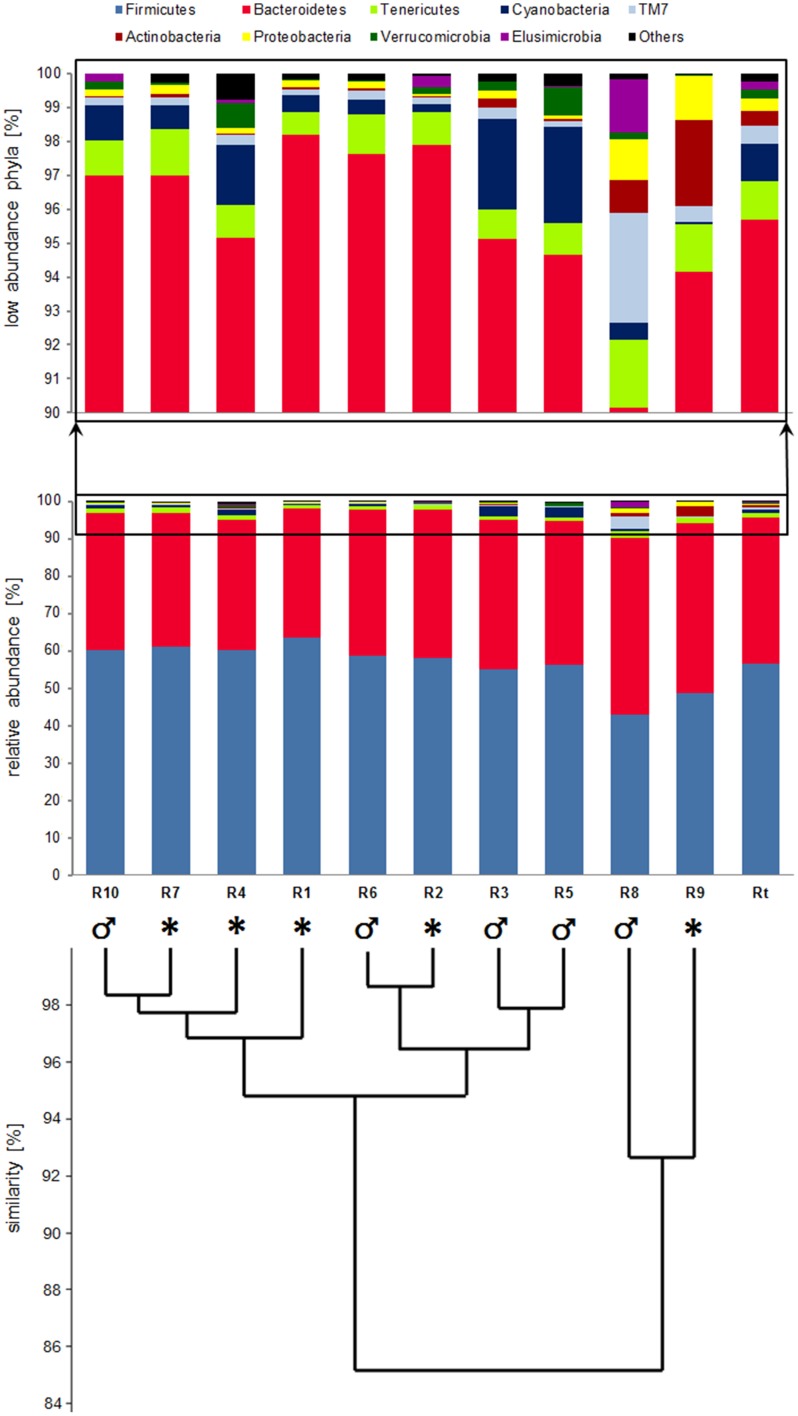
**Abundance of bacterial 16S rDNA sequences at the phylum level**. Analyses of microbial community structure, as well as for each individual (R1–R10) and for total population (Rt). The term “other” corresponds to: *Planctomycetes, Fibrobacteres, Spirochaetes, Chloroflexi, Deferribacteres*. ȷ-male, *-females or young males and females (samples collected from the area where three females and two young individuals were present).

The analysis of fecal bacterial communities in the Svalbard reindeer population from Hornsund showed that 99% of the total reads were represented by *Bacteria* and 1% by *Archaea* (Figure 2). Taxonomy-based analysis showed that fecal bacterial communities consisted of 14 phyla. The most abundant phyla across the population were *Firmicutes* and *Bacteroidetes*, and those phyla jointly accounted for more than 95% of total bacterial sequences (ranging from 90.14 to 98.19%). Separately, *Firmicutes* comprised 56.53% (42.98–63.64%) and *Bacteroidetes* comprised 39.17% (34.56–47.16%) of the total reads (Figures 2, 3). The remaining 5% (on average) of population reads was comprised of *Tenericutes, Cyanobacteria, TM7, Actinobacteria, Proteobacteria, Verrucomicrobia, Elusimicrobia, Planctomycetes, Fibrobacteres, Spirochaetes, Chloroflexi, and Deferribacteres* (Figure 3). SIMPER analysis showed that the microbial community structure in all samples was very much the same (overall similarity for all samples pooled was 92%). The share of *Firmicutes*, followed by *Bacteroidetes*, was primarily responsible for the difference between samples (Table 2). However, the samples did not differ significantly between each other (χ^2^ test with Bonferroni correction, χ^2^ = 67.1, df = 90, *P* = 0.97). Further analysis performed only on the low abundance phyla (10% of the total bacterial sequences) between all of the samples was also not significant (data not shown).

**Table 2:** **Average dissimilarity in microbial community structure**.

**Average dissimilarity (%)**										
	**Rt–R3**	**Rt–R5**	**Rt–R6**	**Rt–R8**	**Rt–R10**	**Rt–R1**	**Rt–R2**	**Rt–R4**	**Rt–R7**	**Rt–R9**
*Firmicutes*	0.8	0.9	1.1	6.8	1.9	3.6	0.8	2.2	2.3	3.9
*Bacteroidetes*	0.7	0.4	0.3	4.0	1.2	2.3	0.4	1.9	1.7	3.1
*Cyanobacteria*	0.4	0.3	0.2	1.3	0.2	0.3	0.3	0.4	0.2	1.0
*Tenericutes*	0.1	0.2	0.1	0.7	0.2	0.2	0.2	0.3	0.2	0.5
*TM7*	0.1	0.2	0.1	0.4	0.1	0.2	0.2	0.2	0.2	0.5
*Actinobacteria*	0.1	0.1	0.1	0.4	0.1	0.2	0.2	0.2	0.1	0.1
*Verrucomicrobia*	0.1	0.1	0.1	0.3	0.1	0.1	0.1	0.1	0.1	0.1
*Elusimicrobia*	0.1	0.1	0.1	0.3	0.0	0.1	0.1	0.1	0.1	0.1
*Proteobacteria*	0.0	0.1	0.0	0.0	0.0	0.1	0.0	0.1	0.1	0.1
Others	0.0	0.1	0.0	0.0	0.0	0.0	0.0	0.1	0.0	0.0
Overall	2.4	2.5	2.2	14.3	3.7	7.1	2.3	5.5	4.9	9.6

*Analysis of abundances of bacterial 16S rDNA sequences at Phylum level, structure estimated for total population (Rt), and each individual (R1–R10). The phylum-specific dissimilarities for each phylum individually and the overall average dissimilarity are presented. Calculated by SIMPER analysis based on Bray-Curtis similarity measure*.

Taxonomy analysis of the population showed that fecal bacterial communities consisted of 30 classes, 46 orders, and 94 families and 141 genera. Two classes: *Clostridia* and *Bacteroidia*, were dominant, accounting for 55.28 and 39.16% of the total reads, respectively (Figure 2). Among the two classes, just one order can be distinguished for each of them, *Clostridiales* and *Bacteroidales*, respectively. Among *Clostridiales*, the dominant family was *Ruminococcaceae*, accounting for 35.17%. Furthermore, 34.30% of the sequences affiliated to the *Bacterioidales* order, belonged to the *Bacteroidaceae* family. With the use of BaseSpace Application 16S Metagenomics v1.0 (Illumina, INC.) we were able to identify the most abundant species in the population, *Bacteroides denticanum*, belonging to the *Bacteroidaceae* family.

Samples from the R8 and R9 individuals were the ones with the most different composition of bacteria, when comparing to the total population (Rt) (Table 2, Figure 3, Supplementary Figure 2). Both individuals were characterized by a similar amount of the two main types of bacteria, *Firmicutes* and *Bacteroidetes* (R8 42.98, 47.16% and R9 48.70, 45.45%, respectively; Figure 3). However, when compared to the total population (Rt), the R8 and R9 individuals exhibited a lower contribution from the *Firmicutes* phylum and higher contribution from *Bacteroidetes* and *Actinobacteria* (Figure 3). R8 also had a higher contribution level from the TM7 phylum. Moreover, R8 had 95.5% of the total reads represented by *Bacteria* and 4.5% by *Archaea* (data not shown). Nonetheless, comparison of each sample (R1–R10) with the total population (Rt), showed that the differences were not statistically significant (χ^2^ tests: χ^2^: 0.93–6.60, df = 9, *P* > 0.05).

## Discussion

This study allows for recognition of bacterial population structure in fecal samples, but does not allow a comparison of rumen and fecal bacterial community structure. However, it has been shown with the use of traditional cultivation methods that most of the dominant bacterial species present in the cecum of Svalbard reindeer were also found at the same ratio in the rumen of the same animal over seasons (Mathiesen et al., 1987). The analysis of fecal bacterial communities presented here and obtained with the use of NGS and 16S rDNA, gives a complex picture when compared to data gained by the use of traditional cultivation methods previously used in this field. Further studies with domesticated ruminants are required to perform a comparative analysis of rumen and fecal bacteria community structures with the use of NGS approaches. That kind of comparison cannot be performed on a wild population.

In the study presented here, at least 800 OTUs, ranking from 800–1002, were observed in each Svalbard reindeer fecal sample, which indicates that the fecal microbial population is highly complex. Rarefication analysis of the obtained data revealed trends indicating that sampling of bacterial communities were almost complete, which could also indicate appropriate efficiency of the DNA extraction method. High values of the Shannon‘s index also suggest a high level of species diversity in the sample. Furthermore, bacterial communities of the tested Svalbard reindeer population are characterized by high and similar values of the Simpson‘s index, considering total reindeer population and each animal separately, which indicates similar diversity in bacterial populations. High number of OTUs, as well as high values of microbial diversity indexes, suggest high representation of species in the tested samples. A high diversity in microbiota communities may be a natural evolutionary strategy for survival associated with seasonal food changes and enable a rapid response to varying food availability at different sites (Henderson et al., 2013) which is crucial considering harsh and extremely seasonal polar conditions and non-migratory lifestyle of the Svalbard reindeer.

High contribution of two phyla (*Firmicutes* and *Bacteroidetes*), comprising more than 90% of the total bacterial sequences in each individual sample, resulted in a lack of significant differences among population. However, the difference among the remaining 10% of strains comprising the bacterial community that includes 12 phyla, despite no substantial impact on the significant differences between individuals, is perceptible. Interestingly, microbial fecal flora of two individuals, R8 and R9, are clustered separately and exhibit the highest diversity. One of the animals is an adult male, which lives rather separately but within tight range of the herd. Given the small size of the studied population, relatively small area utilized by the individuals (during 1 week of the sample collection all individuals were observed grazing within less than 2 km^2^), as well as small travel distances (0.7 km on average) and philopatry of females, the lack of statistically significant differences in the microbial community structure among individuals was expected. Also, between the two tested Svalbard reindeer grazing in their natural winter pastures (Pope et al., 2012) only small variation in rumen bacterial communities has been determined with the use of 16S rRNA gene analysis. The similarity of the microbial structure between individuals may result from the transfer of microbiota within local population, as well as from the fact that animals utilize the same environment and share similar diet composition.

In this study, at the phylum level, *Firmicutes* and *Bacteroidetes* account for more than 95% of the total fecal bacterial community of the Svalbard reindeer. Dominance of these phyla is commonly ascertained in the gut microbiomes of the intermediate ruminant feeders able to digest and utilize a high variety of vegetation, from vascular plants, graminoids, mosses, to lichens, due to highly specialized and unique rumen microbiome (Sundset et al., 2007, 2009, 2010). Also, analysis of rumen bacterial communities in Svalbard reindeer reveals that *Bacteroidetes* and *Firmicutes* were the dominant bacteria, with total share of more than 88% of the total population (Pope et al., 2012). In this study, *Firmicutes* was the dominant phylum with more than 56% share in the total population, and *Bacteroidetes* share was more than 39%, whereas in the other tested Svalbard reindeer the situation was opposite, as *Bacteroidetes* were more abundant than *Firmicutes*, with a share of 61% of the total population (Pope et al., 2012). Such a difference may occur due to many factors, one of them could be the source of the sample (feces and rumen). However, it seems more probable that this difference occurred due to a number of factors, such as sampling period (summer—this study, and winter—Pope et al., 2012) or analysis of different 16S regions, as well as using different extraction methods and data analysis tools.

In studies performed by Sundset et al. (2007) based on 16S rRNA gene library analysis for two sub-species of reindeer, *Rangifer t. tarandus* from northern Norway and *R. t. platyrhynchus* from Spitsbergen, *Firmicutes* also comprised the majority of the bacterial population. For semidomesticated Norwegian reindeer, *Firmicutes* comprised 70.6% and *Bacteriodetes* 29.4% of the total population, with dominant *Clostridiales* and *Bacteroidales* orders, respectively. For another Norwegian reindeer population fed pelleted concentrates, the same phyla were indicated, but with share in the total population of 91.1 and 1.8%, respectively. For Svalbard reindeer, studied by Sundset et al. (2007), those two phyla were also found to be dominant, *Firmicutes* comprising more than 54% and *Bacteriodetes* comprising more than 41% of the total population However, the authors suggest that decrease in the ratio of clones representing *Bacterioidales* in the Norwegian reindeer may be caused by artificial fodder, which may influence the natural gastrointestinal microbial ecology of these animals (Sundset et al., 2007). In our study, *Clostridiales* (>62%) and *Bacteroidales* (>34%) were the most commonly occurring orders, the same as in the other Svalbard reindeer populations (Sundset et al., 2007). Among *Clostridiales*, the most abundant family was *Ruminococcaceae* with the share above 39%, and *Bacteroidaceae* (>31%) was the most abundant family of *Bacteroidales* (Figure 2). With the help of BaseSpace applications, *Ruminococcus* was identified as the most dominant genus among the *Ruminococcaceae* family, and *Bacteroides denticanum* as the most abundant species among *Bacteriodetes*, representing the vast majority of that phylum. *Bacteroides denticanum* was also present in the canine oral microbiome (Dewhirst et al., 2012) and anal and urogenital tract of female Tammar wallaby (Chhour et al., 2008), but still there is insufficient information about abundance and role that it plays in a specific environment. Some bacteria belonging to the *Ruminococcus* genus, described in this study as the dominant genus e.g., *R. flavefaciens, R. albus*, as well as *Fibrobacter succinogenes*, are important in degradation of dietary fiber (Koike and Kobayashi, 2001; Sundset et al., 2007; Singh et al., 2014). Additionally, *R. flavecefaciens* (average share of 0.05%) and *F. succinogenes* present in the samples analyzed in this study, are described as the most commonly isolated cellulolytic rumen bacteria (Koike and Kobayashi, 2001; Sundset et al., 2007). In this study, bacteria belonging to the *Oscillospira* genus, classified within the order *Clostridiales*, were also detected with average share of 0.7% of the total bacterial population. Those bacteria were widely observed under the microscope for almost 90 years and are often found in environmental samples, as well as in the rumen content. However, so far it is impossible to cultivate them as a pure culture (Mackie et al., 2003). The knowledge about their role in these environments is still incomplete.

When studying natural animal populations it is important to gain as much information about them as possible, with interference with their behavior reduced to a minimum. In order to obtain the most consistent description of the tested environment, introducing additional variables during sample collection should be avoided, especially when studying populations with limited or no previous exposure to humans. Fecal sampling can reduce interference with a given population's natural environment and enables to track seasonal and diet changes, and also reduces the cost of testing. It can also reduce the stress which inevitably will accompany collection of rumen samples. The stress itself may influence the composition of the fecal flora and thus falsify the result of the study, as this problem was reported for humans (Holdman et al., 1976). Many factors like antibiotics, buffers, change of diet, environmental stress, and infectious diseases can, inter alia, alter the composition of ruminal ecosystem (Russell and Rychlik, 2001). It was previously reported that environmental stress is one of many factors that can affect methane production in ruminants which is connected with stability of bacterial populations (Kumar et al., 2009). Therefore, collecting and analyzing fecal instead of rumen samples could be considered as a good alternative for any wild animal population, especially those inhabiting natural parks like the Svalbard reindeer.

In conclusion, this paper presents data of fecal bacterial community analysis of endemic Svalbard reindeer *R. tarandus platyrhynchus* population from Hornsund in southwestern Spitsbergen, that was gained using non-invasive testing of a wild population. NGS sequence analysis of the 16S rRNA gene was used instead of traditional cultivation methods, which allowed for identification of a much bigger fraction of bacteria from the digestive tract of Svalbard reindeer. The main implication of this study is that by using non-invasive sampling and modern methods for bacterial identification it is possible to obtain high quality data without any risk to local, sometimes very small, wild populations. Moreover, data gained during this study shows that the population of Svalbard reindeer has a very distinct fecal flora composition from other studied populations, especially of domesticated reindeer. Such information, besides its scientific value, may also be useful for the reindeer breeders, as the microbial diversity of the gastrointestinal tract of livestock is a vital component important for an animals' health and well-being, which is crucial for productivity and many economic aspects.

## Author contributions

SZ performed DNA isolation, sequencing data analysis, wrote the manuscript, took part in planning of the study and discussion; DK performed statistical data analysis, helped in writing the manuscript, and in sample collection; LS, ML, and JL helped in writing the manuscript and took part in planning of the study and discussions.

## Funding

This work was in major part supported by the National Science Center, Poland (grant no. 2011/01/D/NZ2/04817).

### Conflict of interest statement

The authors declare that the research was conducted in the absence of any commercial or financial relationships that could be construed as a potential conflict of interest.

## Supplementary material

Supplementary Figure 1 **Detailed taxonomic analyses on different ranks in the tested feces samples**. Sunburst charts show the relative abundance of bacterial 16S rDNA sequences in total population (Rt) and for each tested individual R1-R10, at different taxonomic levels. The first level of sunburst chart represents all phyla present in particular samples, and the next levels represent class, order, family, genus and species, respectively. html data available at https://www.dropbox.com/sh/elel2ldyr8wb30e/AAD2u6RnFc7K6HJzmfIJBQ6Ga?dl=0 The most suitable browser is Firefox.

Supplementary Figure 2 **Comparison of microbial community with the use of principal coordinate analyses of Bray-Curtis dissimilarities at the phylum level in all tested reindeer individuals**.

Supplementary Table 1 **Detailed taxonomic analysis on different ranks in the tested feces samples**. Table shows the relative abundance of bacterial 16S rDNA sequences for each tested individual R1–R10, at different taxonomic levels.
