# Supplementary material for: New Insights into the Microbiota of the Svalbard Reindeer Rangifer tarandus platyrhynchus
Source: Front Microbiol. 2016 Feb 23;7:170. doi: 10.3389/fmicb.2016.00170 (PMC4763015; doi:10.3389/fmicb.2016.00170)
Supplement: Supplementary Figure 1 — Detailed taxonomic analyses on different ranks in the tested feces samples. Sunburst charts show the relative abundance of bacterial 16S rDNA sequences in total population (Rt) and for each tested individual R1-R10, at different taxonomic levels. The first level of sunburst chart represents all phyla present in particular samples, and the next levels represent class, order, family, genus and species, respectively. html data available at https://www.dropbox.com/sh/elel2ldyr8wb30e/AAD2u6RnFc7K6HJzmfIJBQ6Ga?dl=0 The most suitable browser is Firefox. [file DataSheet2.zip › Supplementary/Supplementary Image 1/R4.html]

Javascript must be enabled to view this page.

magnitude
 1
 1
 4.63733326058489E-04
 0
 0
 0
 0
 2.7278430944617E-05
 2.7278430944617E-05
 0
 0
 0
 0
 0
 0
 0
 0
 0
 0
 0
 0
 0
 0
 0
 0
 0
 0
 0
 0
 0
 0
 0
 0
 0
 0
 0
 0
 0
 0
 0
 0
 0
 0
 0
 0
 2.7278430944617E-05
 2.7278430944617E-05
 0
 0
 0
 0
 0
 0
 0
 0
 0
 0
 0
 0
 0
 0
 0
 0
 0
 0
 0
 4.36454895113872E-04
 4.36454895113872E-04
 4.36454895113872E-04
 4.09176464169255E-04
 2.7278430944617E-05
 0
 0
 0
 0
 0
 .348536512179348
 .348536512179348
 .348536512179348
 7.62977713521749E-02
 .263509642924888
 1.21934586322749E-02
 .251207070568835
 1.09113723778468E-04
 0
 0
 0
 0
 0
 6.54682342670808E-04
 6.54682342670808E-04
 4.61005482964729E-03
 1.4457568400637E-03
 1.4457568400637E-03
 2.01860388990366E-03
 2.01860388990366E-03
 0
 0
 0
 0
 0
 0
 0
 0
 0
 0
 0
 0
 0
 0
 0
 0
 0
 0
 0
 0
 0
 0
 0
 1.78673722686931E-02
 1.78128154068038E-02
 1.78128154068038E-02
 5.4556861889234E-05
 2.7278430944617E-05
 2.7278430944617E-05
 0
 0
 0
 0
 2.7278430944617E-05
 2.7278430944617E-05
 0
 0
 0
 0
 0
 0
 1.06385880683706E-03
 1.06385880683706E-03
 1.06385880683706E-03
 1.06385880683706E-03
 1.06385880683706E-03
 4.91011757003106E-03
 4.91011757003106E-03
 4.91011757003106E-03
 4.91011757003106E-03
 4.91011757003106E-03
 4.91011757003106E-03
 .603126108186806
 3.54619602280522E-03
 2.7278430944617E-05
 0
 0
 0
 0
 0
 0
 2.7278430944617E-05
 2.7278430944617E-05
 2.89151368013441E-03
 0
 0
 0
 0
 0
 2.7278430944617E-05
 2.7278430944617E-05
 2.8642352491898E-03
 2.8642352491898E-03
 6.27403911726191E-04
 6.27403911726191E-04
 6.27403911726191E-04
 .594751629886796
 .594751629886796
 .114160233503728
 4.11904307264017E-03
 4.11904307264017E-03
 7.77435281922086E-03
 2.67328623257647E-03
 5.10106658664438E-03
 0
 1.63670585667702E-04
 1.63670585667702E-04
 5.4556861889234E-05
 5.4556861889234E-05
 6.62320303335551E-02
 3.36343053547218E-02
 4.44638424397157E-03
 1.09113723778468E-04
 5.4556861889234E-05
 2.7278430944617E-05
 .016667121307164
 3.49163916091498E-03
 3.54619602280021E-04
 4.09176464169255E-04
 7.09239204561044E-03
 0
 0
 1.44575684006771E-03
 1.39119997817847E-03
 0
 0
 5.4556861889234E-05
 2.7278430944617E-05
 2.7278430944617E-05
 .39823781336123
 .385171444938754
 5.4556861889234E-05
 5.4556861889234E-05
 8.07441555960864E-03
 5.4556861889234E-05
 4.93739600097868E-03
 1.52759213289655E-03
 6.81960773615425E-04
 6.81960773615425E-04
 0
 1.85493330423696E-03
 1.66398428762464E-03
 0
 1.90949016612319E-04
 4.82828227720523E-03
 4.82828227720523E-03
 4.82828227720523E-03
 4.80100384626061E-03
 2.7278430944617E-05
 2.61872937068123E-03
 2.61872937068123E-03
 0
 0
 0
 2.61872937068123E-03
 2.61872937068123E-03
 1.50031370195494E-03
 8.72909790228746E-04
 2.7278430944617E-05
 8.18352928339512E-04
 2.7278430944617E-05
 0
 0
 0
 0
 2.7278430944617E-05
 2.7278430944617E-05
 0
 0
 0
 0
 0
 0
 0
 0
 0
 0
 0
 0
 0
 0
 0
 0
 0
 0
 0
 0
 0
 0
 3.54619602280021E-04
 3.54619602280021E-04
 3.54619602280021E-04
 3.54619602280021E-04
 2.7278430944617E-04
 2.7278430944617E-04
 2.7278430944617E-04
 1.09113723778468E-04
 1.63670585667702E-04
 0
 0
 0
 0
 0
 0
 0
 0
 0
 0
 0
 0
 0
 0
 0
 0
 0
 0
 0
 0
 0
 0
 2.75512152540932E-03
 2.75512152540932E-03
 2.75512152540932E-03
 2.75512152540932E-03
 9.60200769251631E-03
 9.32922338307014E-03
 8.72909790228846E-04
 8.72909790228846E-04
 8.18352928339512E-05
 7.91074497394895E-04
 8.45631359284129E-03
 2.7278430944617E-04
 2.7278430944617E-04
 7.55612537166392E-03
 3.54619602280021E-04
 3.54619602280021E-04
 3.54619602280021E-04
 1.90949016612319E-04
 1.90949016612319E-04
 1.90949016612319E-04
 7.01055675277158E-03
 7.01055675277158E-03
 7.01055675277158E-03
 7.01055675277158E-03
 5.4556861889234E-05
