# Supplementary material for: New Insights into the Microbiota of the Svalbard Reindeer Rangifer tarandus platyrhynchus
Source: Front Microbiol. 2016 Feb 23;7:170. doi: 10.3389/fmicb.2016.00170 (PMC4763015; doi:10.3389/fmicb.2016.00170)
Supplement: Supplementary Figure 1 — Detailed taxonomic analyses on different ranks in the tested feces samples. Sunburst charts show the relative abundance of bacterial 16S rDNA sequences in total population (Rt) and for each tested individual R1-R10, at different taxonomic levels. The first level of sunburst chart represents all phyla present in particular samples, and the next levels represent class, order, family, genus and species, respectively. html data available at https://www.dropbox.com/sh/elel2ldyr8wb30e/AAD2u6RnFc7K6HJzmfIJBQ6Ga?dl=0 The most suitable browser is Firefox. [file DataSheet2.zip › Supplementary/Supplementary Image 1/Rt.html]

Javascript must be enabled to view this page.

magnitude
 1
 1
 4.1818896117104E-03
 4.55228296991764E-06
 4.55228296991764E-06
 4.55228296991764E-06
 4.55228296991764E-06
 3.41529423758754E-03
 3.41248572216879E-03
 3.31586975263961E-06
 3.31586975263961E-06
 4.55228296991764E-06
 4.55228296991764E-06
 7.41433219715764E-06
 7.41433219715764E-06
 4.44454321207278E-06
 4.44454321207278E-06
 1.4298092713298E-03
 1.96161323346662E-04
 3.31586975263961E-06
 3.31586975263961E-06
 4.55791332188204E-05
 1.28096584851488E-05
 3.18754022085536E-05
 1.00681886757778E-03
 4.55228296991764E-06
 4.55228296991764E-06
 1.28696733770275E-04
 2.09929673558778E-06
 5.25760275366142E-04
 3.12671359847916E-04
 1.51773476854229E-04
 6.13154386639968E-05
 3.10792409910145E-05
 3.02361976729824E-05
 1.03483430155086E-05
 1.03483430155086E-05
 2.5037865407172E-05
 2.5037865407172E-05
 6.52687723149392E-05
 6.52687723149392E-05
 1.46427072985113E-05
 1.00904243285936E-05
 4.55228296991764E-06
 4.55228296991764E-06
 7.03129713977511E-05
 3.58980204538412E-05
 3.44149509439099E-05
 1.05870861602343E-03
 1.05207687651816E-03
 6.63173950526919E-06
 1.19666151670648E-05
 1.19666151670648E-05
 1.49535100823776E-05
 1.49535100823776E-05
 1.59298166928794E-04
 6.63173950526919E-06
 1.52666427423524E-04
 2.09929673558778E-06
 2.09929673558778E-06
 2.09929673558778E-06
 4.55228296991764E-06
 4.55228296991764E-06
 2.80851541875316E-06
 2.80851541875316E-06
 2.80851541875316E-06
 7.33146675170271E-04
 7.33146675170271E-04
 7.33146675170271E-04
 7.09628760296897E-04
 2.35179148733743E-05
 2.88964159826693E-05
 2.88964159826693E-05
 2.60343667554293E-05
 2.86204922724E-06
 2.86204922724E-06
 .391675055740885
 .391630998670668
 .391630998670668
 4.39235418461165E-02
 .343020107342392
 1.93239126756741E-02
 .323559486580601
 1.36708086116466E-04
 2.86204922724E-06
 4.34008940584645E-06
 1.38291594024927E-05
 1.38291594024927E-05
 1.09916691113519E-05
 1.02261468065309E-03
 1.02261468065309E-03
 1.7184259667494E-03
 1.08039692330016E-03
 1.08039692330016E-03
 8.41091082943108E-04
 8.41091082943108E-04
 7.86815272255725E-06
 7.86815272255725E-06
 7.86815272255725E-06
 3.31586975263961E-06
 4.55228296991764E-06
 2.62413082366653E-05
 2.62413082366653E-05
 8.42554625624942E-06
 8.42554625624942E-06
 8.42554625624942E-06
 1.78157619804159E-05
 1.78157619804159E-05
 9.9476092579088E-06
 9.9476092579088E-06
 9.9476092579088E-06
 9.9476092579088E-06
 1.24986530304441E-05
 4.55228296991764E-06
 4.55228296991764E-06
 4.55228296991764E-06
 4.55228296991764E-06
 7.94637006052649E-06
 7.94637006052649E-06
 1.07753135704862E-02
 1.05340432276816E-02
 1.05340432276816E-02
 2.41270342804611E-04
 1.36355230098379E-04
 1.22169891799897E-04
 1.41853382984821E-05
 1.41853382984821E-05
 7.41433219715764E-06
 7.41433219715764E-06
 9.75007805090747E-05
 9.75007805090747E-05
 7.77051109313658E-06
 7.77051109313658E-06
 7.77051109313658E-06
 7.77051109313658E-06
 7.77051109313658E-06
 7.77051109313658E-06
 2.24036493531533E-03
 2.24036493531533E-03
 2.24036493531533E-03
 2.24036493531533E-03
 2.24036493531533E-03
 6.97027593640074E-04
 6.97027593640074E-04
 6.97027593640074E-04
 6.97027593640074E-04
 6.97027593640074E-04
 6.97027593640074E-04
 .565250265230167
 6.21876462141378E-03
 1.57765265935161E-03
 3.13043828297365E-04
 4.55228296991764E-06
 3.08491545327447E-04
 2.1037754797878E-04
 2.69604134503061E-04
 2.69604134503061E-04
 9.95004696551179E-04
 9.95004696551179E-04
 3.08921538170368E-04
 6.63173950527922E-06
 3.31586975263961E-06
 3.31586975263961E-06
 4.55228296991764E-06
 4.55228296991764E-06
 2.7278430944617E-06
 2.7278430944617E-06
 2.9500967260071E-04
 2.9500967260071E-04
 4.33219042389181E-03
 4.33219042389181E-03
 4.33219042389181E-03
 .552837404421656
 .552837404421656
 .130938588849208
 4.6767628179781E-03
 4.6767628179781E-03
 9.40883973111662E-03
 1.50768680043997E-03
 7.88923984931807E-03
 1.1913081358578E-05
 9.16235038138304E-05
 9.16235038138304E-05
 1.25857699493007E-05
 1.25857699493007E-05
 5.25970436879072E-02
 2.87177609176926E-02
 3.46972890511126E-03
 8.82546154793565E-05
 4.46176853300754E-05
 3.641466801283E-05
 1.03432254957236E-02
 4.39104832736759E-03
 2.15390157873979E-04
 2.22988287942479E-04
 5.11013301596791E-03
 5.03078702478955E-06
 2.09929673558778E-06
 8.2373737642974E-04
 7.98230209825833E-04
 1.86254423542791E-06
 1.86254423542791E-06
 2.3644622368479E-05
 1.03838022529478E-05
 1.03838022529478E-05
 .351694884112209
 .34165385417359
 5.4556861889234E-06
 5.4556861889234E-06
 7.02522520167585E-03
 1.32389142194461E-04
 3.01034905075463E-03
 5.15677049986001E-04
 5.0168504044912E-04
 4.99585743713532E-04
 2.09929673558778E-06
 2.08126973034163E-03
 1.88995483733392E-03
 3.96184097101569E-06
 1.87353052036694E-04
 6.19409618709729E-03
 6.19409618709729E-03
 6.19409618709729E-03
 6.13547892148035E-03
 5.86172656169397E-05
 1.54194997400732E-03
 1.54194997400732E-03
 7.86815272255725E-06
 4.55228296991764E-06
 3.31586975263961E-06
 1.53408182128476E-03
 1.53408182128476E-03
 3.91389270123514E-03
 1.54103795552573E-03
 4.80459151279984E-04
 1.00445074374883E-03
 4.42149791383264E-05
 6.56236101188284E-06
 6.56236101188284E-06
 4.55228296991764E-06
 4.55228296991764E-06
 7.81216392773809E-06
 2.7278430944617E-06
 5.08432083327639E-06
 4.14570937423425E-06
 2.04641263864648E-06
 2.09929673558778E-06
 1.12038626754231E-05
 1.12038626754231E-05
 4.55228296991764E-06
 2.80851541875316E-06
 2.80851541875316E-06
 7.13008376037732E-06
 2.80851541875316E-06
 2.22227160603639E-06
 2.09929673558778E-06
 4.55228296991764E-06
 4.55228296991764E-06
 4.55228296991764E-06
 4.55228296991764E-06
 4.55228296991764E-06
 4.55228296991764E-06
 2.80851541875316E-06
 2.80851541875316E-06
 2.80851541875316E-06
 7.73303905338918E-05
 7.73303905338918E-05
 7.73303905338918E-05
 7.73303905338918E-05
 1.18562651113302E-04
 1.18562651113302E-04
 1.18562651113302E-04
 5.5015199728944E-05
 6.35474513843578E-05
 2.17696170406221E-03
 1.48230272270801E-05
 1.48230272270801E-05
 1.48230272270801E-05
 7.41433219715764E-06
 7.41433219715764E-06
 2.86204922724E-06
 4.55228296991764E-06
 2.15262504790239E-03
 2.15262504790239E-03
 1.11840224751868E-05
 2.1414410254272E-03
 1.29837163067394E-04
 3.31586975263961E-06
 2.09929673558778E-06
 2.09929673558778E-06
 2.09929673558778E-06
 1.86254423542791E-06
 1.86254423542791E-06
 1.86254423542791E-06
 1.86254423542791E-06
 1.86254423542791E-06
 5.54030196918164E-03
 5.54030196918164E-03
 5.53807969757561E-03
 5.53807969757561E-03
 1.14708527983859E-02
 1.02292777532536E-02
 1.14092606434714E-03
 1.14092606434714E-03
 1.640958052102E-04
 9.76830259136938E-04
 9.08835168890649E-03
 1.24157504513228E-03
 1.24157504513228E-03
 2.69095416662756E-03
 1.71847782260266E-04
 1.71847782260266E-04
 1.71847782260266E-04
 5.49307608386769E-05
 5.49307608386769E-05
 5.49307608386769E-05
 2.46417562352862E-03
 2.46417562352862E-03
 2.46417562352862E-03
 2.46417562352862E-03
 1.10925433990605E-04
